# Pattern-Induced Visual Discomfort and Anxiety in Migraineurs: Their Relationship and the Effect of Colour

**DOI:** 10.3390/vision6010001

**Published:** 2021-12-24

**Authors:** Trevor J. Hine, Yolande B. Z. White

**Affiliations:** School of Applied Psychology, Griffith University Mt Gravatt Queensland Australia, Mount Gravatt, QLD 4122, Australia; yolande.white@alumni.griffithuni.edu.au

**Keywords:** migraine, visual discomfort, hypervigilance, avoidance, dot-probe tasks, anxiety, colour

## Abstract

In migraineurs, coloured lenses were found to reduce the visual stress caused by an aversive pattern known to trigger migraines by 70%, but do such patterns also produce a low-level anxiety/fear response? Is this response lessened by colour? We sought to investigate this in a study comprising a broad screening component followed by a dot-probe experiment to elicit attentional biases (AB) to aversive patterns. Undergraduate psychology students completed headache and visual discomfort (VD) questionnaires (*N* = 358), thereby forming a subject pool from which 13 migraineurs with high visual discomfort and 13 no-headache controls with low visual discomfort, matched on age and sex, completed a dot-probe experiment. Paired stimuli were presented for 500 ms: aversive achromatic 3 cpd square wave gratings vs control, scrambled patterns. These conditions were repeated using the colour that was most comfortable for each participant. VD was greater in the more severe headache groups. On all measures, the migraineurs were more anxious than the controls, and a positive relationship was found between VD and trait anxiety. The 3 cpd gratings elicited an aversive AB in the migraine group which was somewhat reduced by the use of colour, and this was not seen in the controls. The results suggest a new role for colour in reducing visual stress via anxiety/fear reduction.

## 1. Introduction

### 1.1. Overview

Repetitive, high-contrast, achromatic square wave patterns, repeating at around three cycles per degree (cpd), are known to trigger visual discomfort that can lead to migraines in susceptible people [1]. It is claimed, and has been shown to some extent, that ‘colour therapy’ reduces visual discomfort and decreases the chance of experiencing a visually triggered migraine [2,3]. For instance, colour-tinted glasses have been found to provide therapeutic benefits for both children [3] and adults [4] with visually induced migraines. However, migraines are also associated with anxiety disorders, especially with respect to perceived triggers of migraines where there is a substantial fear component [5,6], while for those with visually triggered migraines, anxiety and neuroticism are associated with visual ‘stress’ or visual discomfort [7]. We tested whether the use of square wave patterns to induce visual discomfort also invokes a pre-conscious aversive response. Additionally, some colours have anxiety-reducing properties and exhibit a calming effect on emotions [8], and we therefore hypothesized that colour may reduce the anxiety and concomitant aversive response. This would suggest a new role for colour therapy for migraines different from that suggested by Wilkins and colleagues [9]. The first aim of the research was to see if visual discomfort was associated with classic migraines (a migraine with aura), especially in terms of the frequency of the migraines. The second aim was to measure if there was an attentional bias (operationalized as dot-probe task reaction times) in migraineurs versus controls towards a high-contrast, achromatic, square wave pattern, repeating at 3 cpd, and whether a colour overlay would reduce this proposed response. The dot-probe task is commonly used to determine the existence of a pre-conscious emotional salience of a particular visual stimulus [10].

### 1.2. Migraines, Anxiety and Fear

Migraines are a headache disorder characterized by intermittent disabling attacks, with various physiological and emotional stressors associated with each episode. With classic migraines, the headache is preceded by 5–60 min of visual aura, e.g., visually perceived light flashes, zig-zag patterns, and geometric colours. The migraine may be aggravated over weeks or months due to psychological stress or environmental factors [11,12,13]. The headaches can be triggered by various stressors, e.g., anxiety, food, bright lights, or certain visual patterns [14]. 

Autonomic nervous system (ANS) dysfunction is a feature in migraines. When individuals face actual or potential stressors, hormonal and neural mediators are activated via the ANS that help to maintain physiological stability (allostasis). These activated responses can become dysregulated when stressors occur often and/or are severe. Load may be accrued through visual exposure to repetitive light–dark borders, which can induce migraines and pattern-sensitive seizures [7,13,15,16]. Migraineurs score highly on stress susceptibility, with high neuroticism being a known correlate of migraines which can lead to panic disorders [6,17]. This is magnified by the pain of a migraine and may become a conditioned emotional fear response to a trigger [18], which can include repetitive high-contrast patterns. The consequence is a pre-conscious judgment of fear-relevant stimuli which originates in the limbic structures [19,20] where the stimulus features that would activate a fear system are aversive, or a pre-existing state of fear or anxiety would need to be present in the patient [21]. In fact, Nulty, Wilkins and Williams [22] investigated the headache component as a possible exacerbating factor of pattern sensitivity in 15 patients aged 30–60 years with high levels of anxiety and depression. These researchers found pattern-sensitivity in significant numbers of people suffering from headaches as a feature of their chronic anxiety and a relationship between their susceptibility to the patterns and their anxiety. 

### 1.3. Colour, Pattern Senstivity and Mood

Precision tinted lenses can minimize the impact of visual triggers in migraines [23,24]. A range of colours provide therapeutic effects for migraineurs (who experience migraines with and without aura) [25]. Using square wave patterns (at 3 and 12 cpd), Shepherd, Hine and Beaumont [25] investigated early visual pathways (achromatic (black/white), tritan (black/purple, black/yellow), protan/deutan (black/red, black/green)) and found colour and spatial frequency to be related to visual pattern sensitivity in migraineurs. Illusions and distortions were reduced when black/colour patterns were presented as opposed to black/white. It is unclear why colour has reduced the symptoms of visual stress, and it was proposed that ‘glare’ plays a major role [5,26,27]. Historically, Wilkins suggested that pattern glare causes a ‘localized hyper-excitability’ of the visual cortex due to a hypersensitivity to contrast, with perceptual distortions occurring through the same mechanisms [15]. Wilkins [15] proposed that colour suppresses this hyper-excitability, preventing the spread of the excitation responsible for the distortions. Using fMRI, Huang et al. [2] have shown that coloured ‘precision ophthalmic tints’ for reducing visual discomfort also reduced cortical activation in area V1 by 5%, and significantly reduced cortical activation in the V2 to V4 areas of the extra-striate cortex by 19% when compared with matched achromatic grey. 

However, apart from colour’s effect at reducing pattern sensitivity, colour also affects mood, particularly anxiety. Jacobs and Suess [28] investigated the emotional effect of the colours red, yellow, green and blue by projecting colours onto a screen, with State-Trait Anxiety Inventory scores as the dependent variable. The highest state anxiety scores resulted from red and yellow. Using physiological measures (e.g., skin conductance response), long-wavelength colours (e.g., red) have been demonstrated to be more arousing than short-wavelength colours (e.g., blue [29]). Valdez and Mehrabian [30] concluded that a blue-purple-rose hue gamut was the least aversive and most calming compared to the green-yellow gamut. In fact, in correctional facilities, aggression- and anxiety-reducing effects have been found when inmates were placed in cells/rooms painted in Baker–Miller pink—a bright, low-saturation, red-purple colour [8,31]. What is unknown is whether the positive effect of the colour on pattern sensitivity in migraineurs is, to some extent, emotional as well as visual. One of the steps in answering this question is to determine whether this affect response is ‘pre-conscious’, and hence not amenable to cognitive moderation. Measuring attentional biases to aversive stimuli using the dot-probe task is a commonly used method in clinical psychology that can help provide an answer to the question [32]. 

### 1.4. Unconscious Assessment of Pattern Anxiety—The Dot-Probe Task 

The dot-probe paradigm reveals how task performance (i.e., detection of the probe) is facilitated or inhibited due to the immediate prior presentation of a stimulus that is related to the concerns (anxiety, fear) of the individual. The reaction time to correctly detect the location of the probe presented in the same location as, but directly after, a priming stimulus see below for detailsis used to measure attentional bias (AB). ‘Hypervigilance’ includes an AB towards threat, increased distractibility, elevated environmental scanning, a wide attentional window prior to threat detection, and a narrow window after threat detection [33,34] leading to a faster reaction time (RT) as opposed to non-threatening stimuli. Research has provided evidence that individuals that score high on trait anxiety have a selective bias towards threatening stimuli, and that a high state anxiety score appears to magnify this bias [33,35]. However, the results of dot-probe experiments depend critically on the duration of exposure to the priming stimulus presented before the probe. Bar-Haim et al. [36] found that timing plays a moderating role in the differences between anxious and control participants for subliminal and 500 ms exposures, with significance not reached for longer exposures. Furthermore, Mogg and Bradley [37] found that in arachnophobes, the hypervigilance found towards pictures of spiders presented for 200 ms significantly decreased with longer exposures (500 ms, 2000 ms). This supports a ‘hypervigilance avoidance’ theory of the dot-probe experiment, whereby the three subcomponents comprising AB are: 1) facilitated attention towards threat (reflecting faster detection of threat-related, compared with neutral stimuli); 2) difficulty disengaging from threat (reflecting slow responses due to slow disengagement from a threat stimulus relative to a neutral stimulus); and 3) attentional avoidance of threat (where attention is allocated towards the location opposite to the threat stimulus location [38,39]). The last subcomponent—avoidance—results in slower RT compared to non-threatening stimuli.

### 1.5. Current Study

There are a number of steps required to achieve the four main aims of the current study. First, to establish whether visual discomfort [40,41] was greatest in migraineurs with aura compared to other headache or no-headache groups and whether the severity of the migraine is related to visual discomfort. Second, to test the link between higher anxiety levels (state and trait [42]) as well as ‘in-the-moment’ distress [43] and visual discomfort in an experimental setting. This was achieved by comparing results from two age- and sex-matched groups, including a migraine group with high visual discomfort and a no-headache group with low visual discomfort. 

The third aim was to measure the unconscious anxiety effects from being exposed to repetitive pattern stimuli using the dot-probe task. The critical measure here is AB towards aversive striped patterns, which, depending on the presentation duration and type of stimulus, can be hypervigilant (faster RTs) or attentionally avoidant (slower RTs). Finally, the last aim was to determine if the effect of therapeutic colour on this AB would be different for the two groups, i.e., migraineurs vs. no headache controls. To achieve this, we would first determine the most and least uncomfortable of four achromatic spatial frequencies flashed under the same conditions as used in the dot-probe task—viewed for 500 ms, in the same screen locations, and under the same lighting conditions. Furthermore, we would determine the most and least uncomfortable colour (colours matched to Wilkin’s Intuitive Overlays; I.O.O. Marketing) under the same conditions as the spatial frequency task. 

## 2. Materials and Methods 

The research was approved by the Griffith University Human Research Ethics Committee: GU Ref No: PSY/H9/07/HRC ‘The Effect of Colour and Visual Noise on Dyslexics and Migraineurs’.

### 2.1. Participants and Screening

Participants (*n* = 356) enrolled in Griffith University’s undergraduate psychology program completed two questionnaires eliciting migraine, headache and visual discomfort data. The questionnaires were the Visual Discomfort Survey (VDS [40]) and a self-report check list for migraines (with and without aura) taken from Section 1 of the International Headache Classification Inventory (IHS [12]). This participant pool consisted of 86 males, 258 females and 12 of no specified gender. Their mean age was 20.87 years and SD 6.05 years. The IHS was used to separate participants into four groups, which are detailed in Table 1, and together with the VDS was used to create two groups for the experimental phase of the research: migraine (U or D) with high visual discomfort (M/HVD) versus no/low headache and low visual discomfort (NoH/LVD). A VDS score of 29 or above was classified as ‘high’, while a score of 16 and below was classified as ‘low’. Based on these screening criteria, sixty-eight people were invited via email to participate in the experimental phase of the study. Thirty-eight agreed to participate, and they were offered one credit participation point and AUD 10, or AUD 15 for travel expenses and their time if they were not first-year students.

Further screening was conducted in the laboratory prior to the experiment. Those who were experiencing, or who had recently experienced (in the last two days) a migraine were excluded. Those who were drowsy due to inadequate sleep or medication were also excluded. Normal or corrected-to-normal vision was required. A test for adequate stereoacuity using the Titmus Fly Stereo-test (Stereo Optical, Chicago, Illinois, [44]) was performed. Finally, normal colour vision was tested using the online Ishihara 38 Plates Colour Vision Deficiency Test (www.color-blindness.com, accessed on 22 May 2015). Those participants with a red-green colour deficiency were also excluded. Each group then comprised 13 participants who were matched for age and sex (M/HVD: male = 1, female = 12, age range: 17–42, M = 22.46, SD = 6.51; NoH/LVD: male = 3, female = 10, age range: 17–35, M = 22.23, SD = 6.27).

### 2.2. Material and Methods 

#### 2.2.1. Materials

1. The VDS comprises 23 items for measuring visual discomfort. The items include questions on perceptual, somatic and performance problems when exposed to different light sources or when reading, as well as severe headache frequency, effective reading times and experiences of visual difficulties whilst reading. Respondents rated answers on a four-point Likert scale (0 = event never occurs, 3 = event occurs almost always). The scores are added. The VDS has good reliability and validity, with a reliable internal consistency estimate of 0.91 [40].
2. The IHS uses self-report diagnostic criteria to obtain data on migraine and headache type, frequency, duration and symptoms, presence of aura and visual discomfort symptoms, along with dyslexia and epilepsy questions.
3. State-Trait Anxiety Inventory—Form Y (STAI [42]). Form Y includes 20 items assessing state and 20 items assessing trait anxiety. Items are rated on a four-point Likert scale (1 = almost never to 4 = almost always) with some items reverse scored. For trait anxiety, participants were required to respond to how they feel generally, and for state anxiety, how they feel right now. The STAI has high internal consistency and reliability (Cronbach’s α between 0.85 and 0.95 [42]) and convergent validity, with high correlations greater than 0.82 at *p* < 0.001 with the Anxiety Sensitivity Index [45].
4. Subjective Units of Discomfort Scale (SUDS [43]). This scale was used to measure moment-to-moment participant anxiety on a self-rating scale from 0 to 10 (0 = not anxious at all, to 10 = extremely anxious), where participants imagine having a ‘distress thermometer’ measuring their anxiety. The SUDS has been validated as global measures of physical and emotional discomfort (Tanner, 2012). The SUDS concurrent validity has been supported with empirical data finding moderate correlations of 0.69 of the SUDS with the STAI [46]. SUDS ratings were conducted at four times during the experiment.

#### 2.2.2. Experimental Apparatus

All stimuli used within the experiment were displayed on a gamma-corrected DELL ST2410B 21” colour monitor, with a 1024 × 768 pixel resolution. The program for the experiment was run using Presentation^®^ software (Neurobehavioral Systems, Inc, Berkeley, CA, USA), with a full-screen refresh rate of 90 Hz. The luminance of the gratings was measured and matched using a Minolta CS-100A Luminance and Colour meter with a 1° probe. The background ambient room lighting around the monitor was 1.2 cd/m^2^. On the monitor, the background grey was 19.2 cd/m^2^. For the achromatic stimulus, the white bars/region were 69.4 cd/m^2^, and the black bars/region were 0.35 cd/m^2^, yielding a Michelson contrast of 99%. All stimuli were presented at a fixed viewing distance of 70 cm.

#### 2.2.3. Experimental Procedure

Following the experimental screening described above, the STAI questionnaires were completed to determine trait and state anxiety. A SUDS rating was completed prior to the experimental phases to establish the baseline measures of current anxiety towards patterns and was repeated at other times during the experiment. 

**Figure 1:**
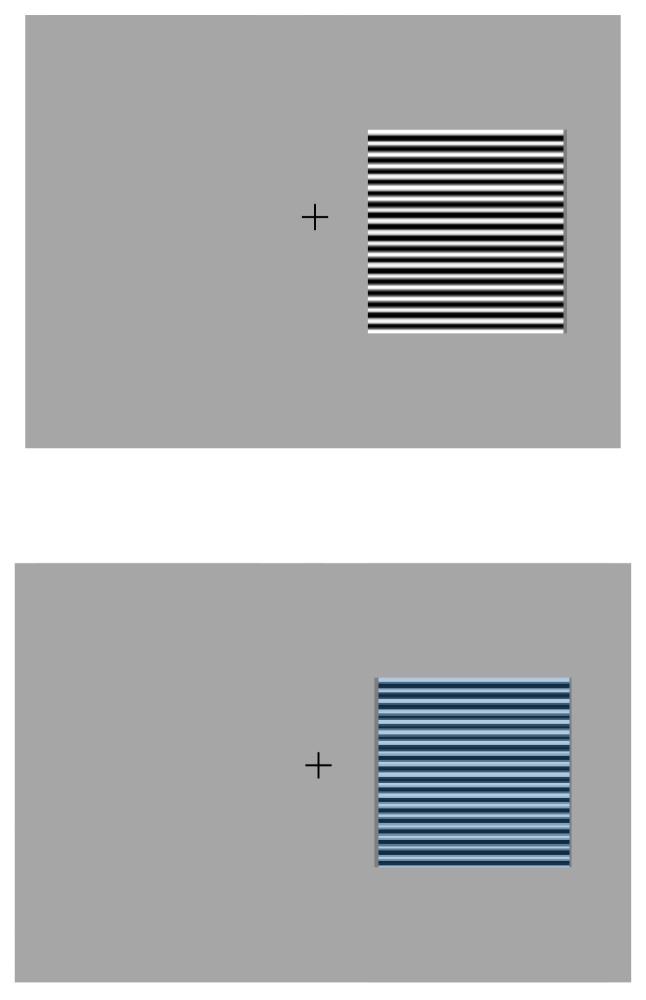
The top image is an example of the type of stimuli displayed in the first phase of the experiment: spatial frequency selection. The bottom image is an example of the type of stimuli used in the second phase of the experiment where each participant’s most uncomfortable spatial frequency was rated when ‘overlain’ with 10 different colours from Wilkin’s Intuitive Overlays.

The experiment itself was conducted in three further phases. Practice trials were presented at the beginning of each phase to accustom participants to each task. The first phase (P1) consisted of the participant selection of the 50% duty cycle, achromatic square wave gratings from 0.5, 3, 5.87 or 11.77 cpd that caused them the maximum or minimum discomfort. Each trial consisted of a 2000 ms grey screen with a central fixation cross (1° per arm) followed by a square region 12° × 12° filled with one of the spatial frequencies presented 1.5° from either the left or right side of the cross for 500 ms (see Figure 1: top). This was then removed, leaving the grey background with five Likert scale choices displayed from left to right (very uncomfortable, uncomfortable, neutral, comfortable and very comfortable). The observer moved the cursor to the left and right using the arrow keys and then pressed the spacebar to record the choice. The responses were converted to digits: ‘very uncomfortable’ = 4, to ‘very comfortable’ = 0. Trials were self-paced. There were 24 trials with six presentations of the four frequencies in a random order. The maximum score possible for any spatial frequency was 24 and the minimum 0. A rest was offered after 12 trials.

The second phase (P2) of the experiment consisted of the participant’s selection of two colour ‘overlays’ that were the most and least comfortable for them digitally placed over their previously selected most uncomfortable spatial frequency. There were ten colours: rose, purple, aqua, lime-green, orange, grey, yellow, mint-green, blue, and pink, all based on Wilkin’s Intuitive Overlays (Institute of Ophthalmology Marketing [47]). All colours were matched for luminance, and each individual colour displayed on the monitor was matched as closely as possible (within the limits of the gamut of the monitor) to the CIE (*x, y*) value of the corresponding I.O.O. overlay (see Figure 1: bottom). The display times and Likert methodology for rating the colours were the same as P1. There were 40 self-paced trials with four presentations of the ten colours in a random order, with rests offered after every 10 trials. The maximum score possible was 16 and the minimum score was 0.

**Figure 2:**
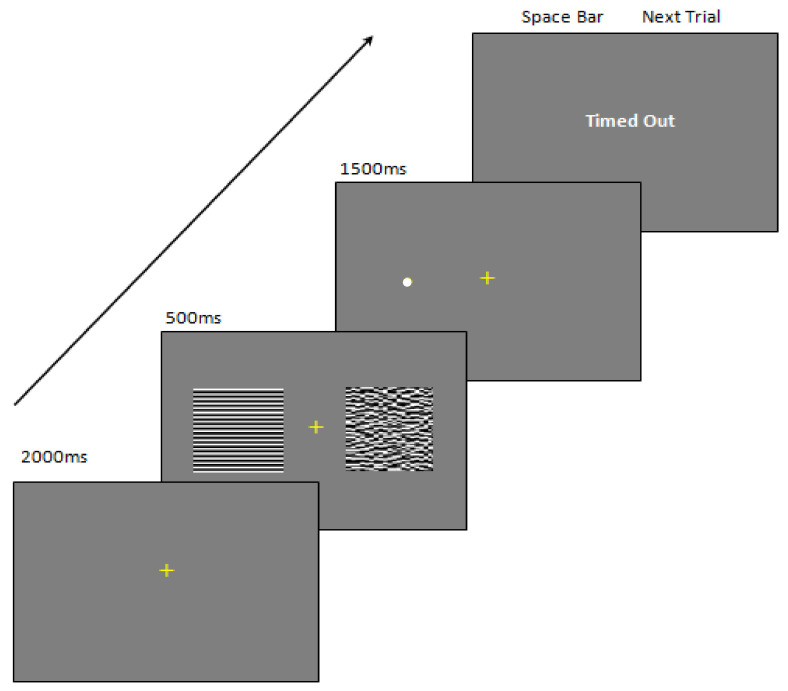
Achromatic dot-probe trial for a stimulus pair of left: 3 cpd, right: scrambled 3 cpd. In the chromatic condition, both stimuli were overlain with one of ten colours matched from Wilkin’s Intuitive Overlays. The pair of stimuli appear for 500 ms side by side. Immediately after, a dot appeared, either on the left or right, centred on the location previously occupied by either stimulus, and participants responded left or right as quickly as possible corresponding to the location of the dot.

The final phase (P3) of the experiment was a dot-probe task. This task was used to test hypervigilance towards an achromatic horizontal square wave 3 cpd (vs. 0.5 cpd) pattern in the M/HVD group compared to the NoH/LVD group, and to determine a reduction in hypervigilance when the pattern was ‘overlain’ with the colour selected by each participant to be the most comfortable for viewing the patterns (see Figure 1: bottom). The 3 cpd gratings were designed to elicit a much greater aversive response than the 0.5 cpd gratings [2,25,48] and this spatial frequency also produced a large and significant difference in comfort ratings between the groups in P1. Additionally, spatially ‘scrambled’ patterns were used as a control non-aversive stimulus (see below). Participants were informed to keep their eyes focused on the fixation cross that would be presented for 2000 ms, after which a stimulus pair would be displayed for 500 ms. Immediately following this, a 0.5° white dot would appear in the middle of where one of the patterns had appeared. The task was to locate the dot as quickly as possible by pressing a left response or a right response key (see Figure 2 for more details). The trials were self-paced. Reaction times were recorded in ms and no response after 1500 ms would result in a time-out and these data were not included for analysis. Participants were informed that breaks could be offered if needed at a quarter, half, and three quarters of the way through the experiment, and that the test would last about seven minutes. 

The stimulus pairs used in P3 are outlined in Table 2. The same three conditions were displayed as either achromatic (see Figure 1: top) or with the preferred colour (see Figure 1: bottom). For both the 0.5 cpd and the 3 cpd, scrambled stimuli were created by dividing each stimulus (12° × 12°) into 1600 ‘tiles’ using a 0.3° × 0.3° mesh, then randomizing the location of each of the tiles within the same area (see Figure 2, second frame from the bottom). The scrambled patterns were used for three reasons. First, disrupting the pattern to reduce the Fourier energy in one direction (vertical) is related to a reduction in visual discomfort [49]. Second, they contained the same spatially averaged luminance as well as areas of black versus white as the intact gratings but did not elicit visual discomfort. Third, if the gratings were to elicit an emotional response, then it was assumed that a scrambled pattern would not elicit as much of an emotional response as it could no longer be identified as a grating. This is analogous to the finding that the emotional effects of faces (e.g., hostile faces) can be differentiated from their low-level visual properties in ‘scrambled’ faces [50,51], where the latter do not elicit an emotional response. Finally, only six conditions were tested and were selected from a potential 28 pairs of stimuli. This was primarily implemented to ensure that the participants, especially in the M/HVD group, did not receive prolonged repetitive exposure to uncomfortable high-contrast grating stimuli. Pilot work using a longer duration experiment led to participants withdrawing from the experiment. For this reason, trials also presented either completely chromatic or achromatic stimuli, and not a mixture of achromatic with chromatic.

There were 12 repeats of each of the six conditions (Table 2). One stimulus was on the left for six trials, and on the right for the other six. Additionally, in six of those repeats, the probe dot was presented on the left, and in the other six, the dot was on the right. The resultant 72 experimental trials were presented in a random order following one minute’s worth of practice. Upon completion, the final SUDS rating was administered. The SUDS rating was also administered before and after P3, to be compared with data gathered before P1 and P2. Participants were debriefed and questioned on their thoughts and feelings experienced during testing.

## 3. Results

For all analyses, unless otherwise stated, the assumptions of linearity and homoscedasticity were met. When the Mauchly’s test of sphericity showed a significant result (*p* < 0.05), a Greenhouse–Geisser correction was always applied to the degrees-of-freedom value.

### 3.1. Analyses of Pool’s VDS and IHS Screening Data

The VDS data were significantly positively skewed (skewness statistic/SE = 5.25) so these data underwent a square-root transformation to remove the skew (skewness statistic/SE = −0.90). One multivariate and one univariate outlier were removed. Significant relationships were found between gender and headache (χ^2^ (1, *N* = 220) = 9.42, *p* = 0.002) and gender and migraine (χ^2^ (2, *N* = 180) = 13.43, *p* < 0.001). Twenty-nine percent of male participants experienced no headaches compared to 12% of the females, 44% of males experienced headaches compared to 49% of the females, and 29% of males suffered from migraines compared to 39% of the females. 

**Figure 3:**
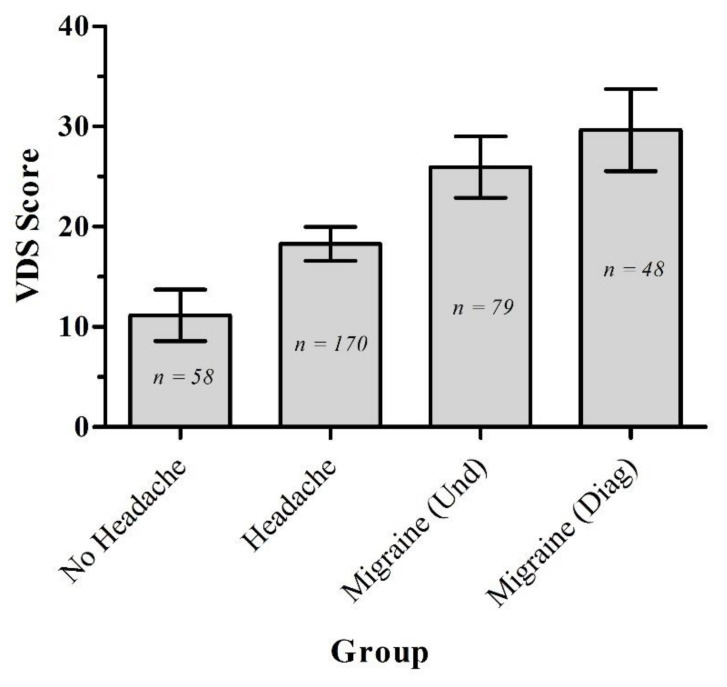
Mean VDS scores for each headache group, with the error bars representing 95% CIs.

**Figure 4:**
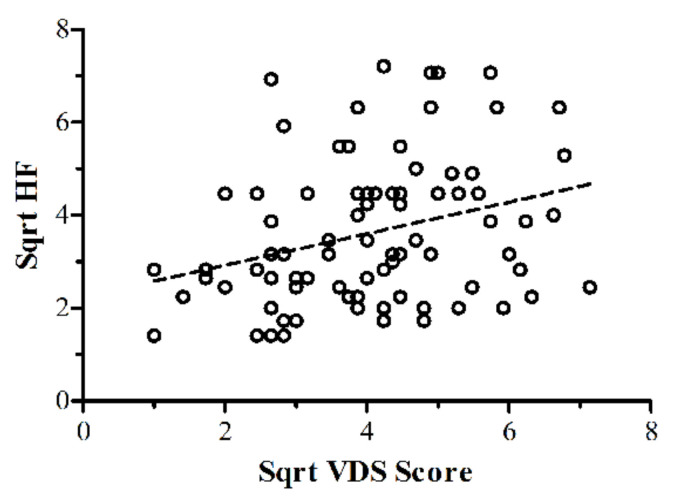
Scatterplot of yearly SqrtHeadache Frequency correlated with SqrtVDS for the migraine headache group showing a correlation of moderate size [52].

The four groups from Table 1 were then compared on their VDS scores (Figure 3). There was a significant effect based on the group: (*F*(3,351) = 30.28, *p* < 0.001, *η*_p_^2^ = 0.21). Bonferroni-corrected (α *=* 0.008) *t*-tests revealed significant differences between all groups, except for migraine undiagnosed vs. migraine diagnosed, with effect sizes ranging from *d* = 0.56 to 1.6 [52]. The severity of headaches was operationalized as ‘average number of headaches a year’, bearing in mind the caveat that it is possible to dissociate between frequent minor headaches with less frequent but more severe experiences. However, we had no access to severity of headache data. The relationship between the headache frequency (where clearly quantitatively reported, *N* = 95) and VDS data was analysed with a Pearson product-moment correlation. These frequency data were skewed (skewness statistic/SE = 11.16), so a square-root transformation was performed to reduce the skew to an acceptable level (skewness statistic/SE = 2.54). Eight outliers were removed. There was a significant positive correlation between SqrtVDS and SqrtHeadache Frequency for these data of moderate effect size (*r* = 0.31, *N* = 87, *p* = 0.004) and this is shown in Figure 4.

### 3.2. Analyses of Experimental Results

An independent samples *t*-test was conducted, ensuring that the groups did not differ on age (*t*(24) = 0.09, *p* = 0.93). No participant was greater than three *SD*s from the mean. A chi-square test was performed that showed no significant differences in sex ratios between the groups (χ^2^(1,26) = 1.18, *p* = 2.77). 

#### 3.2.1. Anxiety

**Figure 5:**
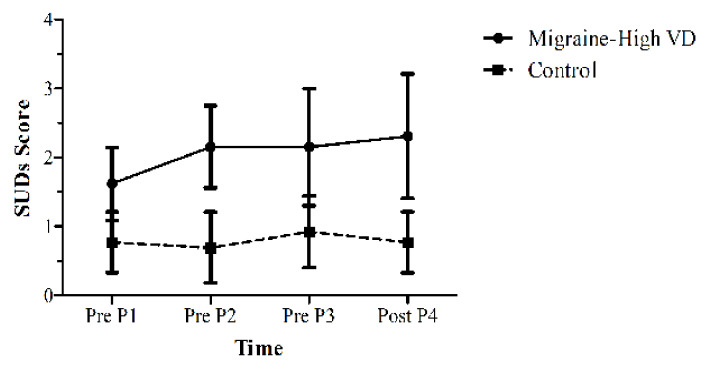
Mean SUDS scores for each group (N = 26, 13 per group) across four points, with three immediately before each test (Pre P1, Pre P2, Pre P3) and one immediately after P3 (labelled Post P4). The error bars represent 95% CIs.

The SUDS data were analysed to determine the effects of various points during the experiment on distress levels. No transformation of these data was required. A mixed model ANOVA was conducted on Time (four levels: PreP1, PreP2, PreP3, PostP4) and Group (two levels: M/HVD, NoH/LVD) against the SUDS score. Figure 5 displays the mean SUDS rating for each group at the various times.

There was a significant main effect of Group (*F*(1,24) = 15.71, *p* = 0.001, *η*_p_^2^ = 0.40), showing a higher distress experienced by the M/HVD group overall, but no interaction with Time (*F*(3,72) = 1.21, *p* = 0.31), nor a main effect for Time (*F*(3,72) = 1.33, *p* = 0.27). In addition, the M/HVD group experienced higher trait anxiety according to the STAI-Y test (*M* = 42.92, *SD* = 12.66) than the control NoH/LVD group (*M* = 33.92, *SD* = 5.82; *t*(16.80) = 2.32, *p* = 0.017, *d* = 1.13). Moreover, the M/HVD group experienced higher state anxiety (*M* = 35.85, *SD* = 10.39) than the control group (*M* = 28.00, *SD* = 5.30; *t*(24) = 2.42, *p* = 0.012, *d* = 0.71). When all experimental participants were included, including those not matched on age and gender, it is interesting to note that there was a significant correlation with a small effect size (*r* = 0.28, *N* = 38, *p* = 0.044, one tail) between VDS and trait anxiety.

#### 3.2.2. Spatial Frequency and Visual Discomfort

The M/HVD group found all spatial frequencies more uncomfortable than the controls. A mixed ANOVA was conducted with Group (M/HVD, NoH/LVD) × Spatial Frequency (four levels: 0.5, 3, 5.9 and 11.8cpd) against the VDS score. The ANOVA yielded a significant main effect of Group (*F*(1,24) = 7.27, *p* = 0.013, *η*_p_^2^ = 0.23) with *t*-tests (α = 0.0125) revealing significant differences between the groups only at 3 cpd (*t*(24) = 2.4, *p* = 0.012, *d* = 0.98) and 5.9 cpd (*t*(24) = 2.62, *p* = 0.008, *d* = 1.07). 

#### 3.2.3. Colour and Visual Discomfort

**Figure 6:**
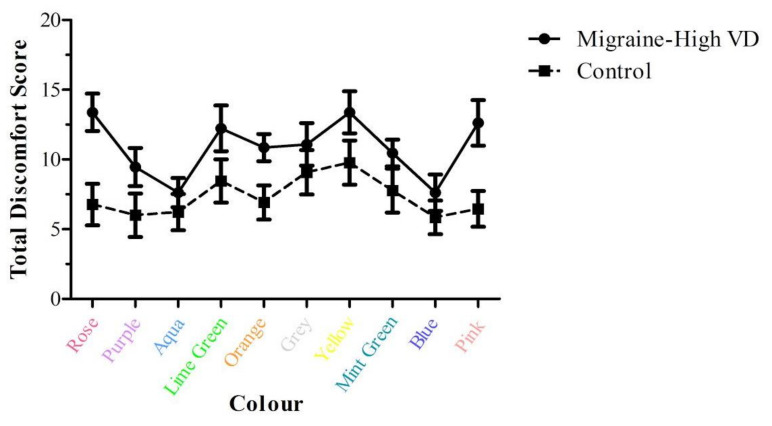
Total Discomfort score for each Group across the ten colours. The error bars represent ±1 SEM (N = 26, 13 per group).

These data are presented in Figure 6. A mixed ANOVA was conducted with Group (M/HVD, NoH/LVD) × Colour (ten levels) against the VDS score. There was a significant main effect of colour (*F*(1,24) = 4.38, *p* = 0.002, *η*_p_^2^ = 0.15). Both groups found aqua and blue to be the least uncomfortable. Since the groups’ discomfort rating diverged for rose and pink, a post hoc exploratory mixed ANOVA was conducted: Group (M/HVD, NoH/LVD) × Colour (four levels: rose, pink, blue, aqua). The analyses revealed that the assumptions of the equality of co-variances and normality were met. There was a significant Group × Colour interaction (*F*(1,1.99) = 3.37, *p* = 0.043, *η*_p_^2^ = 0.12) with a small effect size. The significant interaction was followed up with paired samples *t*-tests looking at the simple effects of the colours between groups (α = 0.008). There was a large significant effect between groups for rose (*t*(24) = 3.3, *p* = 0.002, *d* = 1.35), and for pink (*t*(24) = 2.96, *p* = 0.004, *d* = 1.21). Finally, just within the M/HVD group, there were significant differences (α = 0.025) between each of rose and aqua (*d* = 0.61), and rose and blue (*d* = 0.63).

#### 3.2.4. Dot-Probe Analyses

**Figure 7:**
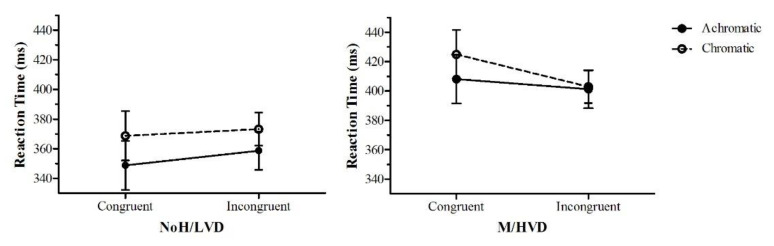
Group, Colour and Congruency effects for conditions one, three, four and six. RTs were averaged over 12 repeat trials for each participant. The error bars represent ±1 *SEM*.

Reaction time (RT) data for correct trials were analysed to determine AB towards the aversive 3 cpd grating and to investigate if colour could reduce this AB. Since the RT data were positively skewed, a geometric mean of the RTs from the trials of each the six conditions (Table 2) was calculated for each participant, and these averaged scores were not skewed. In the following, the term ‘aversive’ refers to the 3 cpd intact pattern, whereas ‘non-aversive’ refers to the scrambled patterns. In addition, probe dots appearing in the location of the aversive 3 cpd pattern were termed ‘congruent’, and dots not appearing in this location were termed ‘incongruent’. A mixed ANOVA was performed that included conditions one, three, four and six (Table 2): Group (M/HVD, NoH/LVD) × Colour (achromatic, chromatic) × Congruency (congruent, incongruent) with averaged RT as the DV. There was a large significant main effect of Colour (*F*(1,24) = 9.06, *p* = 0.006, *η*_p_^2^ = 0.27), and Group (*F*(1,24) = 6.53, *p* = 0.017, *η*_p_^2^ = 0.21) (Figure 7). There were no other significant main effects or interactions; however, there was a trend for the interaction of Colour × Congruency with a medium effect size (*F*(1,24) = 3.51, *p* = 0.073, *η*_p_^2^ = 0.13).

**Figure 8:**
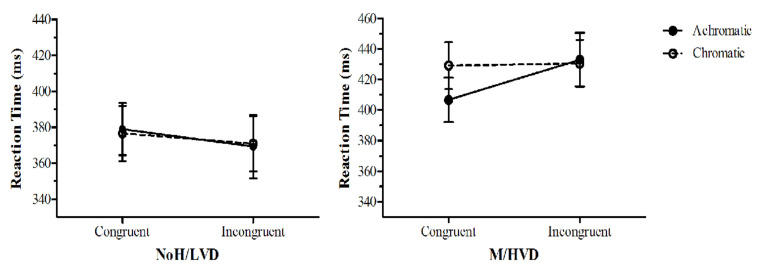
Group, Colour and Congruency effects for control conditions two and five. RTs were averaged over 12 repeat trials. The error bars represent ±1 *SEM*.

Conditions two and five (Table 2) were analysed to determine the relative aversiveness of the two scrambled patterns, as both were assumed to not be aversive compared with the intact 3 cpd pattern. Just in this case, probe dots appearing in the location of the 3 cpd pattern were termed ‘congruent’, and dots not appearing in this location were termed ‘incongruent’. An ANOVA was conducted: Group (M/HVD, NoH/LVD) × Colour (achromatic, chromatic) × Congruency (congruent, incongruent), resulting in a large significant group × colour interaction (*F*(1,24) = 12.25, *p* = 0.002, *η*_p_^2^ = 0.34), and a main effect of Group (F(1,24) = 5.94, *p* = 0.022, *η*_p_^2^ = 0.20). Figure 8 shows that the M/HVD group was faster to respond to dots congruent to the achromatic 3 cpd scrambled pattern than to the achromatic 0.5 cpd scrambled pattern. This difference was not present in the chromatic patterns. The NoH/LVD group’s RTs were all about the same. Bearing this potential confound in mind, an ANOVA was conducted on just conditions one and four, excluding data from trials with the 3 cpd scrambled pattern. There was a main effect for Congruency (*F*(1,24) = 11.05, *p* = 0.003, *η*_p_^2^ = 0.32) and Group (*F*(1,24) = 1788.49, *p* = 0.016, *η*_p_^2^ = 0.22), whereby the M/HVD group’s RTs to dots congruent to the aversive achromatic pattern (mean RT *=* 406.6 ms) were slower than the RTs to the achromatic scrambled pattern (mean RT = 386.7). This effect was not seen in the corresponding chromatic conditions: mean RT = 399.3 vs. *M* = 395.2. The NoH/LVD group did not show this pattern of results for the same conditions: all average RTs were between 368 and 378 ms.

## 4. Discussion

### 4.1. Visual Discomfort and Migraines

From the initial screening data, the higher prevalence of migraine in females over males was in line with previous findings [53]. What was clear though, was that as the severity of the headache typically experienced in each group (Table 1) increased, the level of visual discomfort also increased. This has been demonstrated before in migraineurs [27,54]. However, here we show for the first time that the relationship exists, not just in those who suffer from migraines, but in those suffering from more common types of headaches. In fact, where we had quantitative data on headaches of any kind and their frequency of occurrence, the more frequent the headaches, the more the level of visual discomfort. This supports recently reported results [54] showing a relationship between ‘photophobia’ and headache severity.

### 4.2. Visual Discomfort and Anxiety and Colour

As to the relationship between anxiety and migraines/high visual discomfort, the results from the experiment clearly always show that the M/HVD group had significantly higher trait, state and in-the-moment anxiety than the control NH/LVD group, further supporting the results of Nulty, Wilkins and Williams [22]. Intriguingly, and in line with [22], when all 38 experimental participants were included, a significant correlation was found between trait anxiety and their VDS score (*r* = 0.28, *p =* 0.045).

With respect to the rating of the spatial frequencies producing the most visual stress/discomfort, 3.0 cpd and 5.9 cpd were rated highest; however, 11.8 cpd was also highly rated. These results regarding spatial frequency were not as clear cut as initially reported by Wilkins [15], where 3.0 cpd is reported as clearly the most aversive. The higher spatial frequency could have introduced ‘jazzing effects’ seen in Op Art, a ‘wavy’ motion which is reported as irritating [55], and this, in turn, introduces a confound in the interpretation of the data related to spatial frequency and discomfort. For this reason, and for reasons of consistency across all participants, we resolved to use 3 cpd as the aversive stimulus in the dot-probe P3 phase of the experiment.

Regarding P2 of the experiment, the control group consistently rated some colours as more ‘comfortable’ than others in the blue-purple-pink range. In people suffering from visual stress, Wilkins [15] found that all colours of his Intuitive Overlays were given equal preference. On the other hand, for our M/HVD group under the short presentation time conditions, it was clear that some colours resulted in higher levels of discomfort than others. Pink and rose colours produced the largest difference between the groups, with the migraine group rating them as uncomfortable, while blueish colours were more comfortable for this group. Future studies could explore whether M/HVD require more blue light stimulation, as there is a slight *S*-cone (‘blue-violet’) colour discrimination deficiency in migraineurs [56]. Additionally, further research is needed into the saturation of the colours that prove to be the most comfortable for the M/HVD group, as recent research has shown that migraineurs with aura tend to choose more saturated colours as being comfortable [57].

### 4.3. Unconscious Anxiety Effects and the Effect of Colour in the Dot-Probe Task

The results from this phase of the experiment are not straightforward. Certainly, the M/HVD group had a different pattern of results from the control. Overall, the M/HVD group recorded slower RTs than the control group. M/HVD may have found the entire task to be avoidant, as the exposure duration of 500 ms would have allowed enough time for avoidance to build. In fact, half a second was chosen as the exposure time even though such a duration, rather than say, 100 msec, would most likely produce an avoidant AB [36]. It was reasoned that a very short, nearly subliminal exposure time, while temporally more likely to yield a hypervigilant AB, would not be a long enough exposure for a neural response to occur [15,58]. Alternatively, the achromatic pattern that was flashed for 500ms may have created an illusion of an ‘after-image’ that masked the dot temporarily. Having a higher neural threshold, the NoH/LVD group would be less affected [59].

When the dot is preceded by colour, the overall RTs were slower across both groups. It could be argued that this may be because colour processing is slower than achromatic processing [60]. However, the task on which RTs were measured involved the detection of the same stimuli for both achromatic and chromatic conditions: a white dot. Another possibility is that the colour being present before the detection of the dot calms the participant, slightly reducing arousal levels, and this in turn results in slower RTs.

A comparison of the putatively non-aversive scrambled control patterns revealed that for the M/HVD alone, and not for the control group, there were faster RTs to dots congruent to the 3 cpd scrambled pattern than to the 0.5 cpd scrambled pattern. This difference was reduced from 26 ms to1 ms in the chromatic condition. Clearly, in terms of AB, the 3 cpd scrambled pattern was aversive to M/HVD, but this aversion disappeared with the addition of colour. Why was this the case in what were intended to be the control, non-aversive stimuli? This may be a result of the scrambling having introduced thin line sections, as the first experiment found that the finer gratings were also uncomfortable. 

Because these problems were only evident in the 3 cpd scrambled stimulus, analyses were conducted just on the 3 cpd/0.5 cpd scrambled trials. These partial results did show that the migraine group had a longer RT to the aversive 3 cpd achromatic pattern compared to the scrambled pattern, but the colour gratings and the control pattern showed no such difference. The control group did not show this pattern of results. This does suggest that the 3 cpd grating induced an avoidant AB in the migraine group that was ameliorated by colour. The findings indicate that colour may have the effect of providing better clarity of the image in M/HVD [27]. It is suggested that colour may help identify threats more rapidly, and so the avoidant response could be initiated earlier. This would serve a protective function for the migraineur, shielding them from potential pain, and in this sense it would lower anxiety. The avoidance may be a homeostatic mechanism for down-regulating cortical excitability [61]. Future work should assay 50 msec presentation times so that the avoidance would not have time to build up. 

On the other hand, slower responses to the aversive stimuli could reflect an inability to capture the intervening process because these measures instead allowed the observation of behavioural (rather than cognitive) processes resulting from anxiety. For instance, the automatic vigilance and vigilance avoidance hypotheses propose that, whilst threatening stimuli are visually detected very rapidly due to a secondary process that more thoroughly evaluates the threat, ongoing activity (e.g., the interpretation of emotion) is temporarily interrupted, resulting in a slower behavioural response to threat-related stimuli [62]. It is presumed that anxious individuals direct their attention away from the threat as a strategic attempt to reduce the anxiety elicited by the threatening stimuli. The automatic vigilance and vigilance avoidance hypotheses may account for the disparate findings on attentional processes after threat exposure.

## Figures and Tables

**Table 1:** Four Groups based on Self-report Migraine and Headache Data.

Group	Selection Basis
No Headache	Participants claiming to not experience headaches.
Headache	Participants claiming to experience headaches but not migraines.
Migraine Undiagnosed (U)	Participants claiming to experience migraines (self-diagnosed) and meeting classic migraine criteria (with aura) according to the IHS.
Migraine Diagnosed (D)	Participants claiming to have been diagnosed with migraines (with aura) by a General Medical Practitioner or Neurologist.

**Table 2:** Six stimulus pairs used in the P3: dot-probe experiment.

**Condition**	Position 1 *	**Position 2**	**Colour**
1	0.5 cpd, scrambled	3 cpd	achromatic
2	0.5 cpd, scrambled	3 cpd, scrambled	achromatic
3	3 cpd, scrambled	3 cpd	achromatic
4	0.5 cpd, scrambled	3 cpd	preferred colour
5	0.5 cpd, scrambled	3 cpd, scrambled	preferred colour
6	3 cpd, scrambled	3 cpd	preferred colour

* ‘Position 1’ could be either left or right of the fixation cross, with ‘Position 2’ the opposite location.

## Data Availability

The data presented in this study are available on request from the corresponding author. The data are not publicly available due to participant privacy.
